# Tissue-Region-Resolved Visible–Near-Infrared Hyperspectral Estimation of Nutritional and Structural Traits in *Dendrocalamus brandisii* Shoots

**DOI:** 10.3390/plants15213299

**Published:** 2026-10-26

**Authors:** Haiyang Ren, Xiujuan Zhang, Long Tong, Yanjie Li, Wei Zhang

**Affiliations:** 1 Research Institute of Subtropical Forestry, Chinese Academy of Forestry, Hangzhou 311400, China; 2025721027@yangtzeu.edu.cn; 2 College of Horticulture and Landscape Architecture, Yangtze University, Jingzhou 434025, China; 3 Chongqing Academy of Forestry, Chongqing 401147, China

**Keywords:** hyperspectral imaging, bamboo shoot, nondestructive quality assessment, visible–near-infrared spectroscopy, tissue region

## Abstract

Bamboo shoot quality is determined by sugars, proteins, water status, and cell-wall components that are conventionally quantified by destructive, low-throughput assays. Here we tested whether visible–near-infrared (VIS–NIR) hyperspectral imaging over 400–1000 nm can estimate five nutritional and structural traits—soluble sugar, protein, moisture, lignin, and cellulose—in *Dendrocalamus brandisii* shoots, and whether the tissue region from which spectra are taken matters. Spectra were acquired from 102 shoots, and partial least squares regression (PLSR), Ridge regression, and principal component analysis–Ridge regression (PCA–Ridge) were compared under repeated held-out validation stratified by shoot length class. Under this internal validation, PCA–Ridge gave the highest mean test-set R^2^ for soluble sugar (R^2^ = 0.64 ± 0.04) and lignin (R^2^ = 0.51 ± 0.06), PLSR for protein (R^2^ = 0.62 ± 0.05), and Ridge for moisture (R^2^ = 0.52 ± 0.04). Cellulose was not reliably predictable (R^2^ ≈ 0.04). Modeling the outer and inner tissue surfaces separately showed that the most informative region is trait-dependent: the inner tissue surface provided stronger predictive information for protein and moisture, while the outer surface showed a slight advantage for soluble sugar; for lignin, the regional difference was small. Within length classes, soluble sugar was positively correlated with hardness in the 30 cm class (r = 0.728, BH-adjusted *p* = 0.005). VIS–NIR hyperspectral imaging combined with regularized regression thus offers a workable route to estimating soluble sugar, protein, moisture, and lignin (cellulose was not estimable in the 400–1000 nm window) from a single acquisition in *D. brandisii* shoots, with trait-specific ROI placement. Models were trained on a single sampling batch that included six shoots of other bamboo species, and require independent *D. brandisii*-only validation across sites, seasons, and instruments.

## 1. Introduction

*Dendrocalamus brandisii* (Munro) Kurz is a sympodial bamboo distributed across several geographic populations in Yunnan, China, which together retain appreciable genetic diversity [1]. The species is valued both for its edible shoots and for its timber, but its naturally tall stature sits awkwardly with the limited headroom of protected cultivation facilities. Topping has been shown to regulate plant architecture, growth, and shoot quality in *D. brandisii*, and therefore offers a route to dwarfed production under cover [2]. Protected cultivation is also attractive because sympodial bamboos in southern China can suffer severe frost injury during extreme cold and snow or ice events, with marked differences among species [3]. Dwarfing, environmental adaptation, and quality regulation consequently need to be addressed together if greenhouse production of *D. brandisii* is to be viable.

Shoot quality itself is a composite target. Sugars, amino acids and proteins, water status, fiber, and the progress of lignification jointly determine palatability, processing behavior, and storage stability [4,5]. Rapid measurement of these traits would allow shoots to be graded at harvest for fresh-market or processing use, and would help identify shoots prone to rapid postharvest lignification and texture loss before storage [5]. Conventional physicochemical assays quantify these components reliably, but they consume the sample, take hours to days, and cannot be scaled to the number of shoots a breeding or grading program must screen. A shoot destroyed to measure its sugar content can no longer be planted, sold, or resampled, which is precisely the bottleneck that nondestructive phenotyping is meant to remove.

Spectroscopic sensing offers an alternative. Hyperspectral imaging resolves spectral and spatial information simultaneously, whereas near-infrared spectroscopy characterizes absorption and reflectance rapidly at the point of measurement; both are now routine for nondestructive evaluation of fruit and vegetable quality and safety [6,7,8]. In the 400–1000 nm window, VIS–NIR reflectance arises mainly from visible pigment absorption and from the relatively weak second O–H stretching overtone of water near 970 nm, with indirect contributions from carbohydrate and cell-wall status via scattering and tissue-structure covariance [7]. Hyperspectral prediction therefore serves as a complementary high-throughput screening tool rather than a replacement for standard wet-chemical reference assays. In bamboo shoots, near-infrared spectroscopy has been applied to nondestructive hardness measurement and to model optimization [9,10]. Previous bamboo shoot studies have mainly focused on classification, freshness, or individual quality traits. What remains largely untested is whether a single VIS–NIR acquisition can support estimation of several nutritional and structural traits at once, and whether the choice of tissue region—the tough outer surface as opposed to the soft tissue near the shoot core—changes how much predictive information the spectrum carries [11]. Because a shoot is anatomically heterogeneous and its composition shifts rapidly during elongation, this choice is unlikely to be neutral.

We therefore combined VIS–NIR hyperspectral imaging with regularized regression to phenotype *D. brandisii* shoots across a length gradient. Our objectives were to (1) build spectral estimation models for soluble sugar, protein, moisture, lignin, and cellulose and compare three regression approaches; (2) test whether the outer surface and the inner tissue surface differ in the predictive information they provide for each trait; (3) rank shoots by predicted trait value and examine the spectral and spatial characteristics of the resulting candidates; (4) describe how the five traits vary across shoot length classes; and (5) examine the correlation structure between these traits and hardness, and among the traits themselves. The study is intended as a methodological basis for rapid quality screening and high-throughput phenotyping of bamboo shoots; the internal validation reported here is not evidence of precision cultivation or of causal regulatory mechanisms.

## 2. Materials and Methods

### 2.1. Experimental and Technical Design

**Figure 1:**
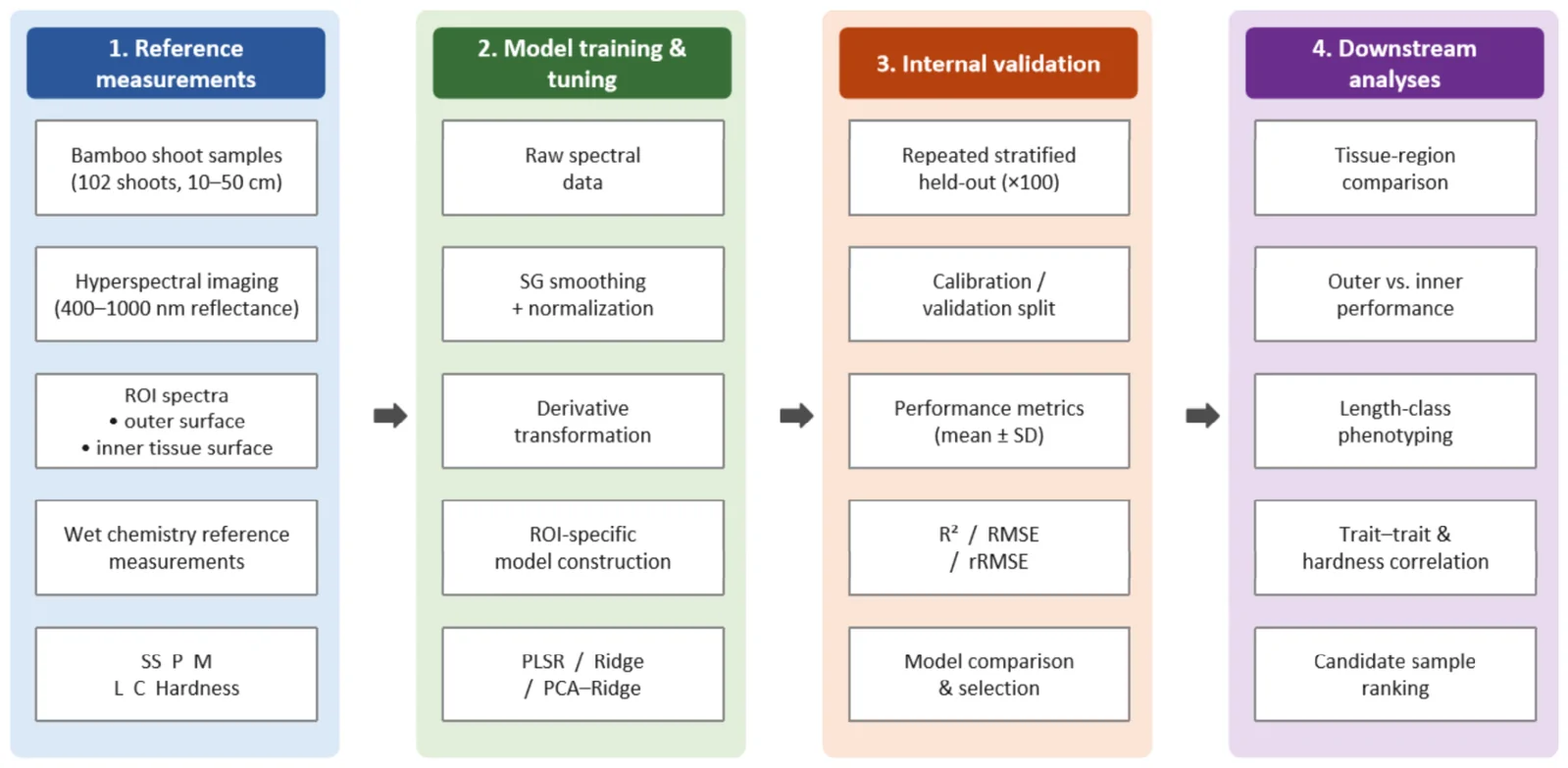
Experimental and analytical workflow for hyperspectral estimation of nutritional and structural traits in bamboo shoots. The workflow comprises four stages: (1) reference measurements, including hyperspectral imaging (400–1000 nm) of 102 shoots across 10–50 cm length classes, extraction of spectra from outer-surface and inner-tissue-surface regions of interest (ROIs), and wet-chemical determination of soluble sugar, protein, moisture, lignin, cellulose, and hardness; (2) model training and tuning, including spectral preprocessing and fitting of partial least squares regression (PLSR), Ridge regression, and principal component analysis–Ridge regression (PCA–Ridge); (3) internal validation using 100 repeated stratified held-out splits, with performance summarized as mean ± SD of the coefficient of determination (R^2^), root mean square error (RMSE), and relative RMSE (rRMSE); and (4) downstream analyses comprising tissue-region comparison, length-class phenotyping, pairwise trait and trait–hardness correlations with Benjamini–Hochberg (BH) correction for multiple testing, and high-prediction candidate sample ranking. SS, soluble sugar; P, protein; M, moisture; L, lignin; C, cellulose; ROI, region of interest; PLSR, partial least squares regression; PCA, principal component analysis; BH, Benjamini–Hochberg.

The workflow comprised shoot sampling and reference-trait measurement, VIS–NIR hyperspectral imaging and extraction of region-of-interest (ROI) spectra, spectral preprocessing and regression modeling, model evaluation and candidate identification, and length-class trait comparison with exploratory correlation analysis (Figure 1).

### 2.2. Study Area

**Figure 2:**
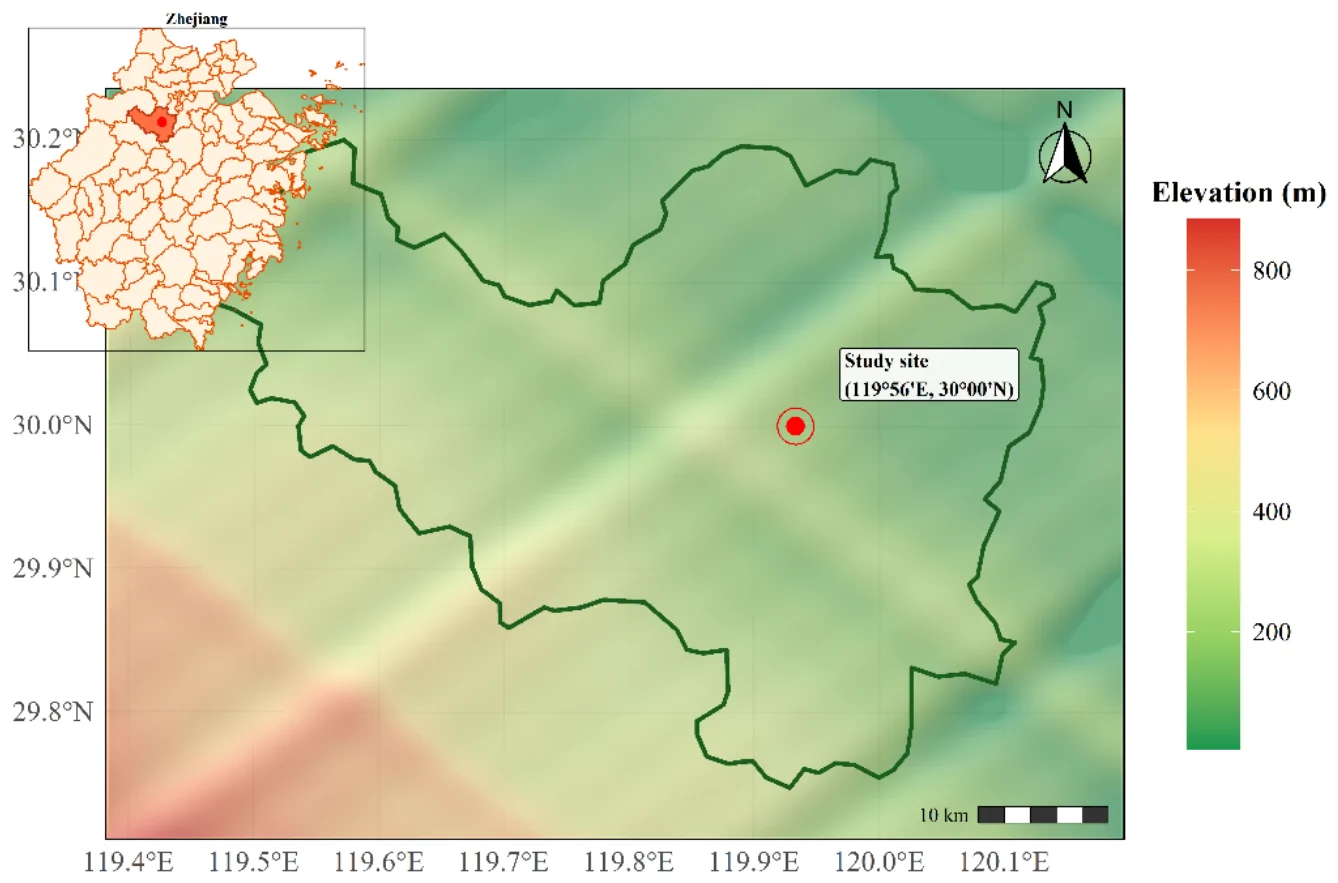
Study area. The main map shows the topography of Fuyang District, Hangzhou, Zhejiang Province, China, with the experimental site (119°56′ E, 30°00′ N) marked by the red circle. The principal *D. brandisii* material was collected from this Fuyang greenhouse; additional reference bamboo shoots were obtained from a field nursery in Anji, Zhejiang. The inset (upper left) shows the position of Fuyang District within Zhejiang Province.

Figure 2 shows that the study site was at Shangxintiao Village, Jiangbin Village, Lushan Subdistrict, Fuyang District, Hangzhou, Zhejiang Province, China (119°56′ E, 30°00′ N). The region has a subtropical monsoon climate with cold winters, hot summers, distinct seasons, abundant precipitation, and adequate sunshine; annual mean relative humidity is 70.3%, annual precipitation is 1454 mm, and annual sunshine duration is 1765 h. The experimental area covered approximately 1 ha inside an EVA-film greenhouse with 90% light transmittance. *D. brandisii* was transplanted into the greenhouse from Zhangzhou, Fujian Province, in spring 2020 by root-ball transplantation at 2 m × 1.5 m spacing between and within rows. The greenhouse was fitted with remote temperature and humidity monitoring (DYF20A) and drip irrigation, and irrigation and ventilation were used to moderate high summer temperatures and keep the growing environment stable.

### 2.3. Data Acquisition

#### 2.3.1. Indoor VIS–NIR Hyperspectral Image Acquisition

**Figure 3:**
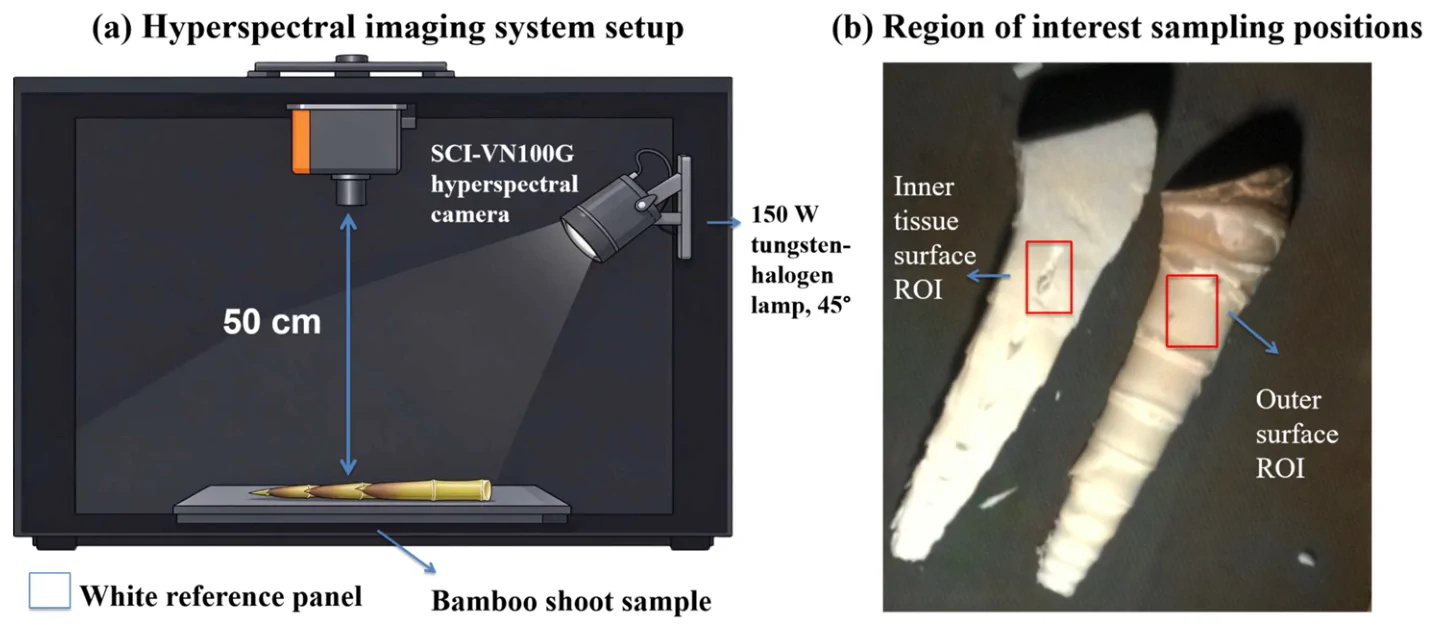
Hyperspectral imaging system setup and region-of-interest sampling positions. (**a**) Schematic of the dark box imaging system. The SCI-VN100G hyperspectral camera (Westlake Intelligent Vision Technology (Hangzhou) Co., Ltd., Hangzhou, China) was mounted vertically 50 cm above the sample stage, with a 150 W tungsten-halogen lamp positioned at 45° on the right inner wall. Bamboo shoots were placed horizontally on the sample stage, with a Spectralon white reference panel adjacent for radiometric calibration. (**b**) Photographs showing the regions of interest (ROIs). Red boxes indicate the approximately 200 × 200 pixel ROIs on the outer surface and the inner tissue surface of longitudinally cut shoots.

Images were acquired with a SCI-VN100G VIS–NIR hyperspectral camera over 400–1000 nm (≥100 spectral channels, maximum spectral resolution 3 nm, spatial resolution 1024 × 1024 pixels). The camera was mounted vertically inside a dark box with its lens 50 cm above the sample stage (lens-to-stage distance; Figure 3a), and the integration time was 10 ms. Illumination was provided by a single 150 W tungsten-halogen lamp mounted at 45° on the right inner wall of the dark box. White and dark reference calibration was performed before each imaging session: a Spectralon white reference panel was scanned to obtain the white frame, and the lens was capped to acquire the dark frame. Relative reflectance was then calculated as R = (I_raw − I_dark)/(I_white − I_dark), where I_raw, I_white, and I_dark are the raw sample, white-reference, and dark-reference intensities, respectively. All shoots were imaged on 1 June 2025. Outside conditions averaged approximately 20.5 °C and 97% relative humidity; greenhouse ventilation and shading moderated internal temperatures. Each shoot was placed horizontally in the center of the dark box with the sampled surface facing the camera, and imaging was performed immediately after excision to minimize water loss. The outer surface was imaged first; the shoot was then cut longitudinally to expose the inner tissue surface, which was imaged within 2 min of cutting.

#### 2.3.2. Plant Materials, Length Classes, and Standard Phenotyping

The *D. brandisii* shoots were collected from 12 mother clumps in the Fuyang greenhouse, representing shoots at 3–10 days after emergence. Samples comprised *D. brandisii* shoots grown in the Fuyang greenhouse together with shoots of other bamboo varieties collected from a field nursery in Anji, Zhejiang. In total, 102 shoots spanning a length gradient of approximately 10–50 cm were collected, covering approximately days 3–10 after emergence. Within each of the 12 mother clumps, several shoots at 3–10 days after emergence were harvested (approximately 8 shoots per clump on average, giving 96 *D. brandisii* shoots in total), so that clump (genet) and shoot (ramet) form a two-level hierarchy. The sample size was not fixed in advance: all shoots from the 12 clumps that fell within the 10–50 cm range at sampling were harvested, and the six reference shoots brought the total to 102. For trait comparison and correlation analysis, the 96 *D. brandisii* shoots were assigned to five length classes—nominally 10 cm (10–15 cm), 20 cm (15–25 cm), 30 cm (25–35 cm), 40 cm (35–45 cm), and 50 cm (45–50 cm)—which correspond to successive developmental stages from approximately day 3 to day 10 after emergence and contained 16, 20, 19, 19, and 22 shoots, respectively. The remaining six shoots (IDs c1–c6), which belonged to bamboo varieties other than *D. brandisii*, were retained as reference material and were not assigned to any length class. Length differences among the 96 *D. brandisii* shoots reflect the days since emergence rather than different growth environments: all shoots came from the same greenhouse, were sampled on the same day, and spanned emergence days 3–10, so variation in light, temperature, and precipitation over this short interval is negligible relative to the developmental gradient. During the 3–10 day emergence window, the greenhouse temperature averaged approximately 22 °C (range 18–28 °C) and relative humidity approximately 90%, maintained by ventilation and shading.

Reference values for the five nutritional and structural traits and for hardness were obtained destructively, with three measurements averaged per sample in every case. Soluble sugar was determined by the copper-reduction iodometric method (NY/T 1278-2007), protein by the Kjeldahl method (GB 5009.5), and moisture by oven drying at 105 °C to constant mass (GB 5009.3). Lignin was determined according to GB/T 10337-2008 and cellulose by the nitric acid–ethanol method [12]. Hardness was measured by puncture testing with a texture analyzer at the testing facility of the Research Institute of Subtropical Forestry, Chinese Academy of Forestry; the maximum penetration force (N) was recorded at three evenly spaced positions on the middle section of each shoot. All measurements followed the relevant national standards and were completed within a single batch to keep the reference data internally consistent. Soluble sugar, protein, and moisture are expressed on a fresh-weight basis, and lignin and cellulose on a dry-weight (oven-dried) basis. Reference measurements showed the following ranges: soluble sugar 0.22–1.93 g/100 g, protein 1.50–3.16 g/100 g, moisture 92.0–94.3% (g/100 g fresh weight), lignin 7.02–31.7 g/100 g (dry basis), cellulose 12.5–30.2 g/100 g (dry basis).

#### 2.3.3. ROI Definition and Spectral Reflectance Extraction

Prior to imaging, all culm sheaths were removed by hand to expose the edible culm surface. The outer-surface ROI was placed on the exposed culm epidermis at the midpoint of the shoot. After the outer-surface spectrum was acquired, the shoot was cut longitudinally to expose the inner culm tissue, and the inner-tissue-surface ROI was placed on the freshly cut face near the shoot center. A rectangular ROI of approximately 200 × 200 pixels was placed at the center of the sampled surface, aligned with the wet-chemical measurement position (Figure 3b). ROIs were delineated in ENVI 5.3 (Harris Geospatial Solutions, Boulder, CO, USA) and mean reflectance spectra were extracted from the hyperspectral images. ROIs were placed as close to the sample center as the shoot allowed, away from the image margins, to limit vignetting near the lens edge. Two ROI types were extracted per shoot: the outer surface, meaning the tough outermost surface of the edible culm, and the inner tissue surface, meaning the soft tissue close to the shoot core. Because the inner surface requires cutting and is therefore not fully non-destructive, this study compares tissue-region differences to support future outer-surface nondestructive detection.

### 2.4. Machine-Learning Regression Models

#### 2.4.1. Whole-Sample Modeling

Regression models were implemented in R 4.4.1 (R Foundation for Statistical Computing, Vienna, Austria) using the pls and glmnet packages. Whole-sample spectra were preprocessed and modeled with three regression methods: partial least squares regression (PLSR), Ridge regression, and principal component analysis–Ridge regression (PCA–Ridge).

(1) PLSR. Partial least squares regression extracts latent variables aligned with the response and is widely used in chemometrics for high-dimensional predictors with strongly correlated bands [13]. The number of latent variables (n_components) was treated as a hyperparameter, and values were compared under a repeated stratified held-out framework; combinations that were stable across repeated validations were preferred.
(2) Ridge regression. Ridge adds an L2 penalty to the linear regression loss and shrinks coefficients toward zero, which limits the parameter instability and variance inflation that highly correlated predictors otherwise produce [14]. The regularization coefficient α was the main hyperparameter and was optimized by grid search with cross-validation to balance bias against variance. Ridge does not require bands to be discarded beforehand, since shrinkage itself controls the influence of redundant variables—a property well matched to spectra in which adjacent wavelengths are strongly correlated.
(3) PCA–Ridge regression. PCA–Ridge first projects the spectral variables onto mutually orthogonal principal components by principal component analysis (PCA) and then fits a Ridge model to the component scores. Retaining components on explained predictor variance alone can discard information that is relevant to the target trait but carries little variance [15]. The number of components (n_components) and the regularization coefficient α were therefore optimized jointly by cross-validation rather than fixing the component number from cumulative explained variance.

Spectral preprocessing was fitted independently within each training split to avoid data leakage. Seven methods were evaluated: (i) Savitzky–Golay first derivative (window size = 7); (ii) area normalization, dividing each spectrum by the sum of its absolute reflectance values; (iii) linear detrending, retaining the residual from a regression of reflectance on wavelength index; (iv) absorbance conversion (−log10 of reflectance); (v) sample-wise Min–Max normalization; (vi) standard normal variate (SNV) transformation; and (vii) multiplicative scatter correction (MSC) referenced to the training-set mean spectrum. Savitzky–Golay smoothing and differentiation suppress noise and baseline drift while preserving local spectral shape, whereas normalization and detrending reduce the effects of scale, scattering, and baseline variation on model fitting [16,17].

Feature-engineering settings for the Ridge models were likewise trait-specific: soluble sugar (top-k = 12 bands, log1p-transformed response), protein (top-k = 28 bands, square-root-transformed response), moisture (top-k = 16 bands, untransformed response), lignin (top-k = 6 bands, log1p-transformed response), and cellulose (top-k = 8 bands, untransformed response). The Ridge regularization coefficient (λ) was searched over 0.01–1000 for every trait. PLSR models were tuned over 2–10 latent variables, and PCA–Ridge models over 5–30 principal components with λ again searched over 0.01–1000. Top-k bands were selected within each training fold by the absolute Pearson correlation between each band and the response in that fold; the k bands with the largest |r| were retained and held fixed for the test fold. The value of k and the response transformation (none, log1p, or square root) were likewise chosen within the training data, together with λ, by cross-validation; the held-out test set was not used for these choices.

#### 2.4.2. Region-Specific Modeling of the Outer Surface and Inner Tissue Surface

The outer surface and the inner tissue surface differ in texture, chemical composition, and scattering behavior, so the two ROI types were extracted and modeled separately. The outer surface is the tough outermost surface; the inner tissue surface is the soft tissue close to the shoot core. Both data sets were processed with raw spectra, absorbance conversion, area normalization, Min–Max normalization, and derivative transformations. PLSR, Ridge, and PCA–Ridge were all evaluated on both region datasets, with PLSR tuned over 2–10 latent variables as in Section 2.4.1.

The procedures were as follows:

(1) Ridge regression. Separate Ridge models were fitted to the outer-surface and inner-tissue-surface data, with the regularization parameter optimized by parameter search. Hyperparameters were tuned within the training split, and preprocessing–model combinations were compared on their mean performance across the same 100 repeated stratified held-out runs used for the whole-sample models (Section 2.4.3).
(2) PCA–Ridge regression. PCA was used to reduce the dimensionality of the spectral data and a Ridge model was fitted to the component scores, with the number of components and the regularization parameter searched jointly. As for the Ridge models, these comparisons are relative rather than an unbiased estimate of external generalization.

Because region-specific preprocessing and model combinations were screened in the same way as the whole-sample models, the region-specific R^2^ values are likewise internal-validation results; they are used for relative comparison between tissue regions and are not directly comparable to the whole-sample R^2^ values, which were obtained from different spectra.

#### 2.4.3. Model Selection and Validation Strategy

Splits were performed at the sample level (not pixel level), with approximately 80 training and 20 test samples per trait. Feature selection (top-k bands and response transformation) was conducted exclusively within the training folds, without accessing the held-out test set. The data were split 80:20 into training and test sets by stage-stratified sampling, so that every length class was represented proportionally in both sets. All 102 shoots with reference measurements, including the six reference-variety shoots, entered model construction, and a fixed random seed (seed = 2026) was used throughout for reproducibility. Model stability was assessed by evaluating all candidate combinations with 100 repeated stratified held-out runs. The training set was used for fitting and the test set for evaluation, and the final model for each trait was chosen on performance and stability across the 100 repeated runs rather than on a single split. Because preprocessing and model combinations were screened alongside test-set evaluation, the reported test-set R^2^, RMSE, and rRMSE are best read as relative comparisons under these experimental conditions; their implications for generalization are taken up in Section 4.5. We acknowledge that comparing preprocessing-model combinations within internal validation may introduce optimistic bias, and test-set R^2^ can exceed training-set R^2^ under small sample sizes. Reported performance should be interpreted as internal validation results rather than independent external generalization.

### 2.5. Model Performance Evaluation

Model performance was quantified by the coefficient of determination (R^2^), root mean square error (RMSE), and relative RMSE (rRMSE), following standard chemometric practices [18].

R^2^ is measured against a baseline that predicts the mean reference value, so on an independent test set it can fall below 0 when a model performs worse than that baseline. RMSE carries the unit of the corresponding trait, whereas rRMSE normalizes RMSE by the mean reference value and is expressed as a percentage, allowing errors to be compared across traits.

### 2.6. Prediction and Identification of High-Prediction Candidate Samples

The better-performing models identified in Section 2.4.1 and Section 2.4.2 were used to generate predicted values of the five traits for every shoot. Inputs used the same hyperspectral data and preprocessing as during model construction, and the models were applied separately to the whole-sample, outer-surface, and inner-tissue-surface data. Performance continued to be judged on test-set R^2^, RMSE, and rRMSE under the split described above; full-sample predictions served only for sample ranking and candidate identification and were not treated as independent validation.

Shoots were then ranked separately by predicted soluble sugar, protein, moisture, lignin, and cellulose, and the five highest-ranking shoots for each trait were retained as high-prediction candidates, with sample ID, predicted value, and associated hyperspectral information recorded. Outer-surface and inner-tissue-surface predictions were ranked separately so that the distribution of candidates across tissue regions could be compared. These rankings are model outputs and do not by themselves confirm that a shoot is genuinely high in a trait or represents superior germplasm.

To characterize the candidates in spectral space, reflectance from the corresponding ROIs was visualized in R 4.4.1 (R Foundation for Statistical Computing, Vienna, Austria). Representative candidates were selected for each of the five traits and mapped as spatial distributions of mean reflectance, with color encoding reflectance level so that spectral responses could be compared within and between candidates.

### 2.7. Statistical Analysis

Trait distributions were compared across the five length classes (10, 20, 30, 40, and 50 cm) by computing the mean, standard deviation, and range of soluble sugar, protein, moisture, lignin, cellulose, and hardness for each class, and by displaying the distributions as boxplots.

Linear associations among the five traits, and between each trait and hardness, were examined by Pearson correlation within each length class. Coefficients (r) were computed for all ten pairwise trait combinations and for every trait–hardness pair, and assembled into correlation matrices. Coefficients range from −1 to 1, with the sign giving the direction of the association and |r| its strength.

Because multiple tests were performed within each class, *p*-values were adjusted by the Benjamini–Hochberg (BH) procedure, and a BH-adjusted *p* < 0.05 was taken as significant. All correlation analyses were run independently within each length class.

## 3. Results

Model performance evaluation was confirmatory, while candidate sample ranking, trait correlation analysis, and length-class phenotyping were exploratory.

### 3.1. Whole-Sample Model Performance

**Figure 4:**
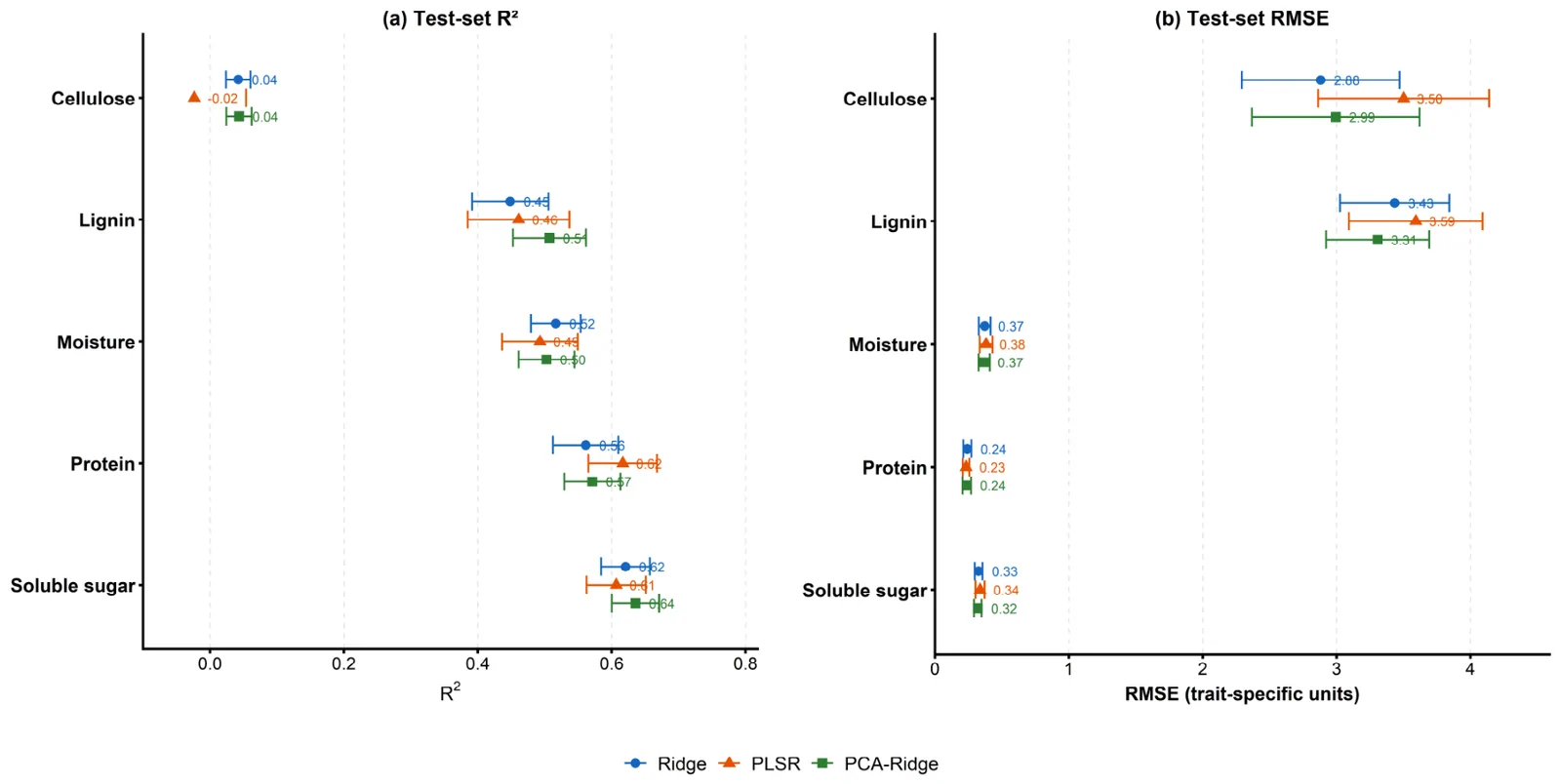
Predictive performance of three models (Ridge, PLSR, and PCA–Ridge) across five bamboo shoot traits. (**a**) Dot plot of test-set R^2^ for the three models across the five traits; traits are listed on the y-axis and R^2^ on the x-axis, with points further right indicating better prediction. (**b**) Dot plot of test-set RMSE for the three models across the five traits; traits are listed on the y-axis and RMSE on the x-axis, with points further left indicating lower error. Blue circles, orange triangles, and green squares denote Ridge, PLSR, and PCA–Ridge, respectively. Error bars represent ±SD. All metrics were computed on the test set only. PLSR, partial least squares regression; PCA–Ridge, principal component analysis–Ridge regression. Values are means ± SD across 100 repeated stratified held-out splits; these represent internal validation performance, not independent external test performance. RMSE units are trait-specific (g/100 g for soluble sugar, protein, lignin, and cellulose; % for moisture).

Ridge, PLSR, and PCA–Ridge were compared on test-set R^2^ and RMSE for the five target traits (Figure 4).

Across 100 repeated stratified held-out runs, no single model dominated all traits. PCA-Ridge achieved the highest mean test-set R^2^ for soluble sugar (0.636 ± 0.035, RMSE = 0.320 ± 0.028 g/100 g) and lignin (0.507 ± 0.055, RMSE = 3.306 ± 0.385 g/100 g, dry basis); PLSR performed best for protein (R^2^ = 0.617 ± 0.051, RMSE = 0.232 ± 0.025 g/100 g); and Ridge performed best for moisture (R^2^ = 0.517 ± 0.037, RMSE = 0.372 ± 0.044%). Cellulose was not reliably predicted by any model (best R^2^ = 0.042 ± 0.018, RMSE = 2.880 ± 0.590 g/100 g, dry basis). All values are means ± SD across 100 repeated splits and represent internal validation performance (Section 2.4.3). The relatively wide spread in lignin RMSE across model–preprocessing combinations reflects its weaker spectral signal: with R^2^ ≈ 0.5, small differences in preprocessing or shrinkage substantially affect the fitted prediction, whereas the better-predicted traits show tighter RMSE distributions. Model performance was trait-dependent. Soluble sugar showed the highest R^2^ (≈0.64), followed by protein (R^2^ ≈ 0.62), moisture (R^2^ ≈ 0.52), and lignin (R^2^ ≈ 0.51); cellulose was essentially unpredictable (R^2^ ≈ 0.04). No single regression method was uniformly superior across all five traits. rRMSE, calculated as RMSE divided by the mean reference value, was approximately 32% for soluble sugar, 10% for protein, 0.4% for moisture, 18% for lignin, and 14% for cellulose. Because R^2^ describes the proportion of variation explained whereas RMSE and rRMSE describe prediction error on each trait’s own scale, the two criteria do not rank the traits identically: soluble sugar had the highest R^2^ but also the largest relative error, whereas moisture had a moderate R^2^ but a very small rRMSE, reflecting its narrow range (92.0–94.3%) and high mean. Traits are therefore compared here on R^2^ together with RMSE and rRMSE, not on R^2^ alone.

### 3.2. Region-Specific Model Performance of the Outer Surface and Inner Tissue Surface

**Figure 5:**
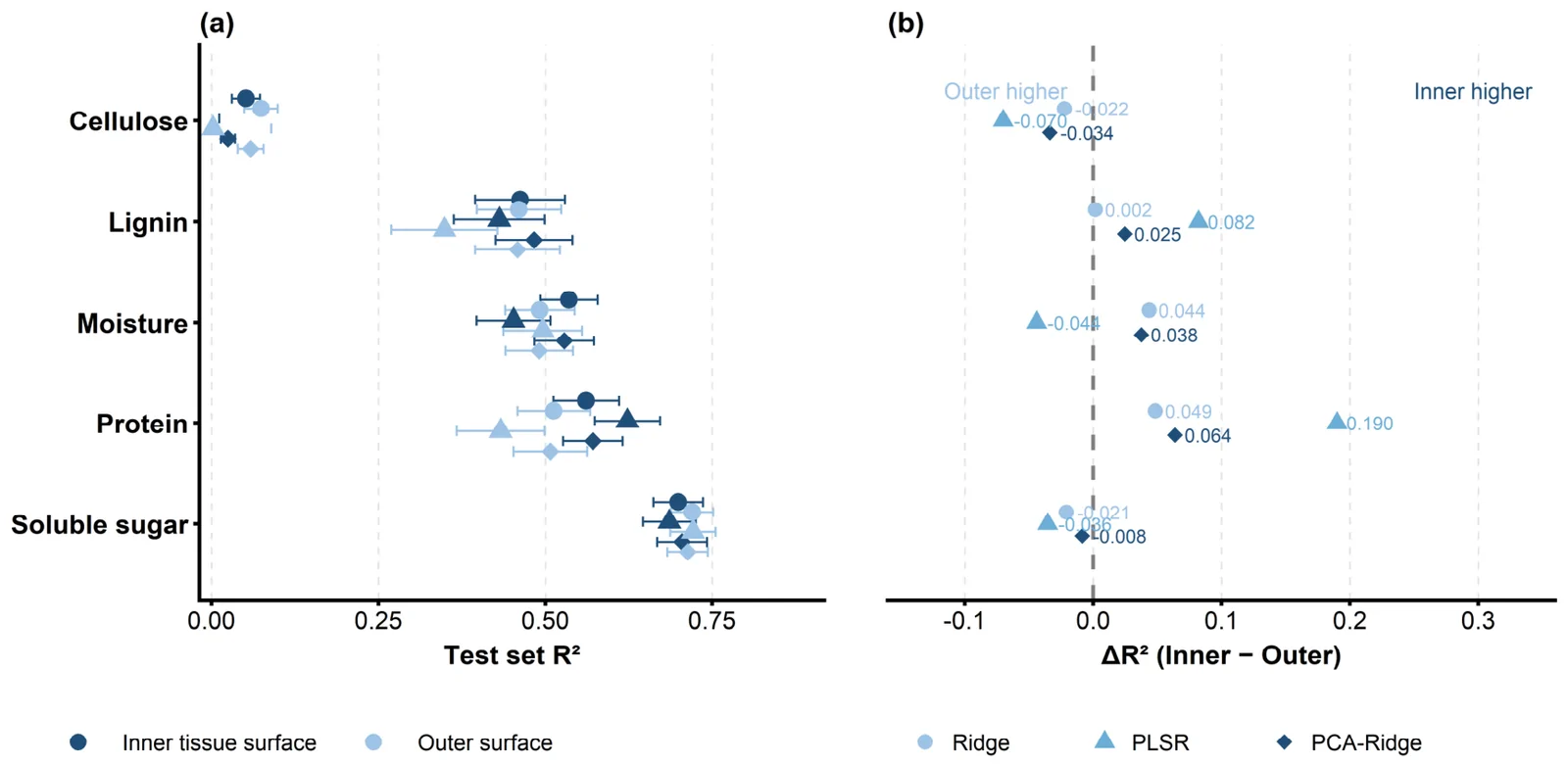
Effects of tissue region and regression model on test-set prediction performance for five bamboo shoot traits. (**a**) Test-set R^2^ obtained from outer-surface and inner-tissue-surface spectra using Ridge, PLSR, and PCA–Ridge. (**b**) Difference between regions, calculated as ΔR^2^ = R^2^(inner) − R^2^(outer); positive values indicate better prediction from the inner tissue surface and negative values from the outer surface. Each symbol is one tissue-region–model–trait combination. Error bars represent ±SD. PLSR, partial least squares regression; PCA–Ridge, principal component analysis–Ridge regression. Values are means ± SD across 100 repeated stratified held-out splits; these represent internal validation performance, not independent external test performance.

PLSR, Ridge, and PCA–Ridge models were then built separately from outer-surface and inner-tissue-surface spectra and evaluated on test-set R^2^ and RMSE (Figure 5). Performance varied with both trait and tissue region. No single model was uniformly best across all region–trait combinations. PLSR performed best for soluble sugar on the outer surface and for protein on the inner surface; Ridge performed best for moisture on the inner surface; and PCA–Ridge performed best for soluble sugar and lignin on the inner surface.

For soluble sugar, the outer surface (PLSR R^2^ = 0.721 ± 0.034, RMSE = 0.266 ± 0.027 g/100 g) was comparable to the inner surface (PCA-Ridge R^2^ = 0.705 ± 0.037, RMSE = 0.269 ± 0.028 g/100 g). Protein showed the largest regional gap, with the inner-tissue-surface PLSR model reaching R^2^ = 0.623 ± 0.049 and RMSE = 0.226 ± 0.026 g/100 g compared with 0.513 ± 0.055 and 0.248 ± 0.030 g/100 g for the best outer-surface model (Ridge). For moisture, the inner tissue surface provided stronger predictive information (Ridge R^2^ = 0.536 ± 0.043 vs. outer PLSR R^2^ = 0.496 ± 0.059; RMSE 0.361 ± 0.045% vs. 0.385 ± 0.047%). Lignin was estimated slightly better from the inner tissue surface (PCA-Ridge R^2^ = 0.483 ± 0.058, RMSE = 3.383 ± 0.427 g/100 g, dry basis) than from the outer surface (Ridge R^2^ = 0.461 ± 0.063, RMSE = 3.428 ± 0.459 g/100 g, dry basis). Cellulose remained poorly predicted from both regions (best R^2^ ≈ 0.05–0.07, RMSE ≈ 2.9–3.2 g/100 g, dry basis).

The informative region is therefore trait-dependent. The inner tissue surface provided stronger predictive information for protein and moisture, the outer surface showed a slight advantage for soluble sugar, and for lignin the regional difference was small. Where the ROI is placed thus affects hyperspectral estimation, and it affects each trait differently.

### 3.3. High-Prediction Candidate Samples and Their Spectral Characteristics

**Figure 6:**
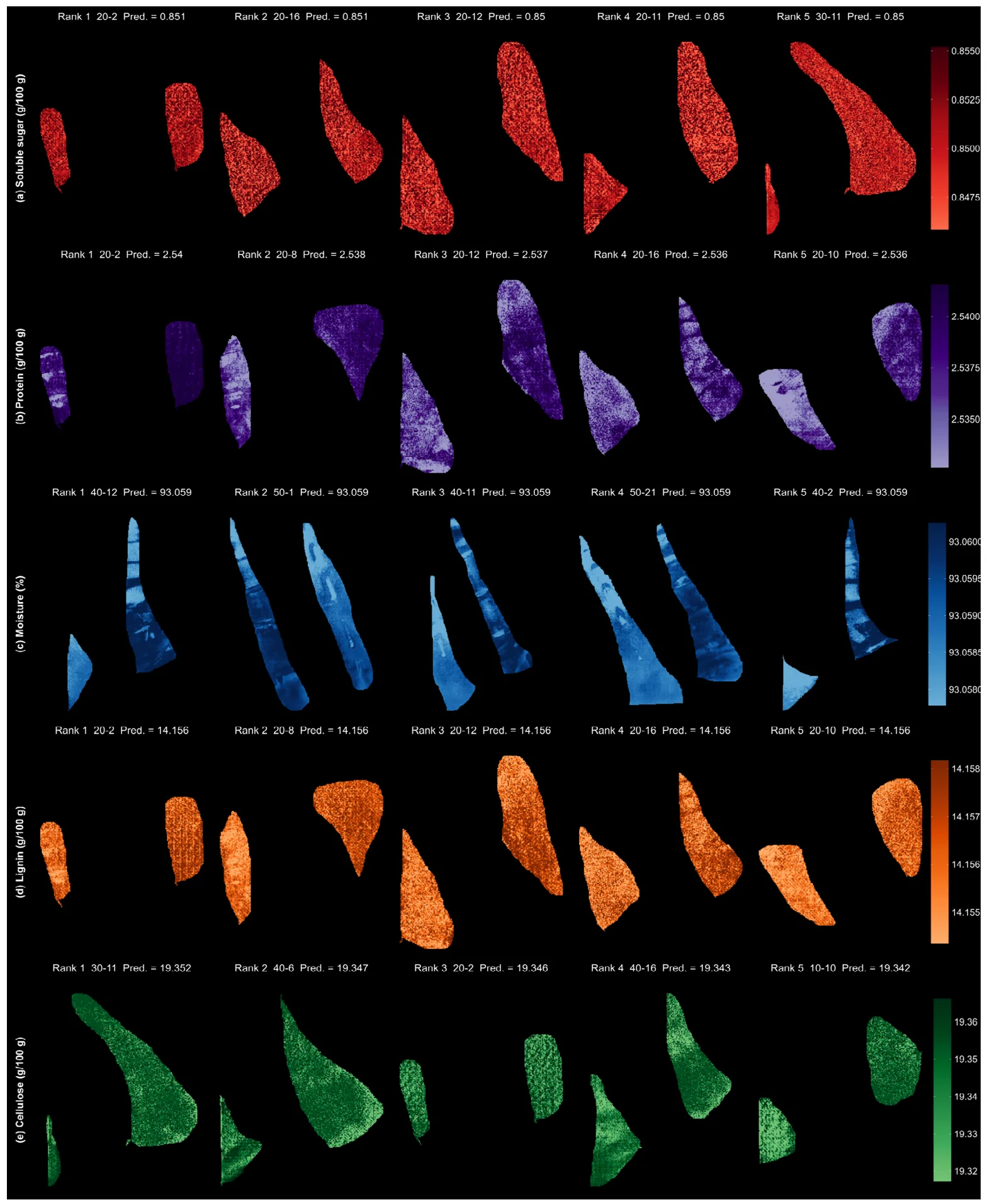
Pixel-wise predicted distributions of five traits in the five highest-ranking *Dendrocalamus brandisii* shoots for each trait. Shoots were ranked on sample-level predictions from the selected hyperspectral models, with Rank 1 to Rank 5 shown left to right. (**a**) Soluble sugar (g/100 g); (**b**) protein (g/100 g); (**c**) moisture (%); (**d**) lignin (g/100 g, dry basis); (**e**) cellulose (g/100 g, dry basis). Pred. is the sample-level predicted value used for ranking, whereas pixel colors give the corresponding local predicted values. Within each trait, a common color scale across the five ranked shoots allows predicted levels and within-sample spatial heterogeneity to be compared directly.

Shoots were ranked by predicted value for each trait and the five highest-ranking shoots were retained as candidates, giving 16 unique shoots once duplicates across traits were removed. These shoots are useful targets for remeasurement, but their predicted values still need confirmation by independent wet-chemistry analysis and repeated sampling. Pixel-wise predicted distributions for the five candidates per trait are shown in Figure 6.

### 3.4. Trait Variation Across Shoot Length Classes

**Figure 7:**
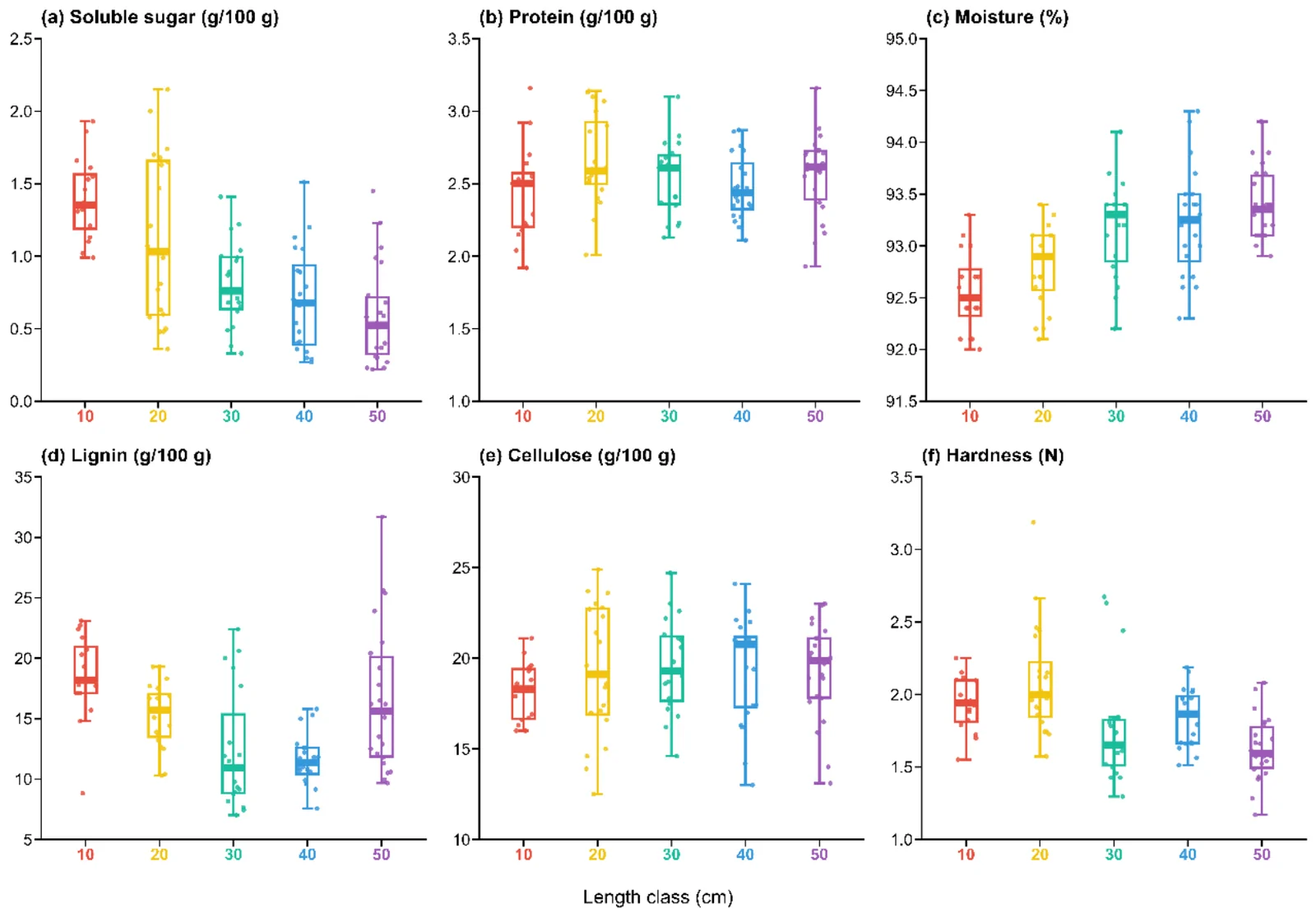
Distribution of nutritional and structural traits and hardness across bamboo shoot length classes. Boxplots show (**a**) soluble sugar (g/100 g), (**b**) protein (g/100 g), (**c**) moisture (%), (**d**) lignin (g/100 g, dry basis), (**e**) cellulose (g/100 g, dry basis), and (**f**) hardness (N) in the 10-, 20-, 30-, 40-, and 50 cm classes. The center line is the median and the box limits the first and third quartiles; whiskers extend to the most extreme observations within 1.5 × the interquartile range (IQR), and individual shoots are overlaid as jittered points. The analysis included 96 shoots, with *n* = 16, 20, 19, 19, and 22 for the 10-, 20-, 30-, 40-, and 50 cm classes, respectively.

Soluble sugar, protein, moisture, lignin, cellulose, and hardness were compared across the 10-, 20-, 30-, 40-, and 50 cm classes (Figure 7), and the traits showed somewhat different distributions. Soluble sugar appeared lower in the 50 cm class than in the 30 cm class, and moisture appeared higher in longer shoots. Protein varied little across classes. Lignin appeared lower in the 30- and 40 cm classes and more variable in the 50 cm class, whereas cellulose changed little overall. Hardness fluctuated across classes. The wider spread of soluble sugar and cellulose within the 20 cm class (*n* = 20; Figure 7a,e) may coincide with the onset of rapid internode elongation; this is reported descriptively and was not tested formally. No formal between-class statistical test was performed; these are descriptive observations from the boxplots. These contrasting backgrounds motivate examining trait–hardness and trait–trait relationships within each class separately.

### 3.5. Exploratory Correlation Analysis Between Traits and Hardness Across Length Classes

Pearson correlations between model-predicted trait values and measured hardness were computed within each length class, with BH correction for multiple testing [19,20,21]. Because the trait values entering these correlations are model predictions rather than direct measurements, the associations below describe patterns rather than establish relationships.

**Figure 8:**
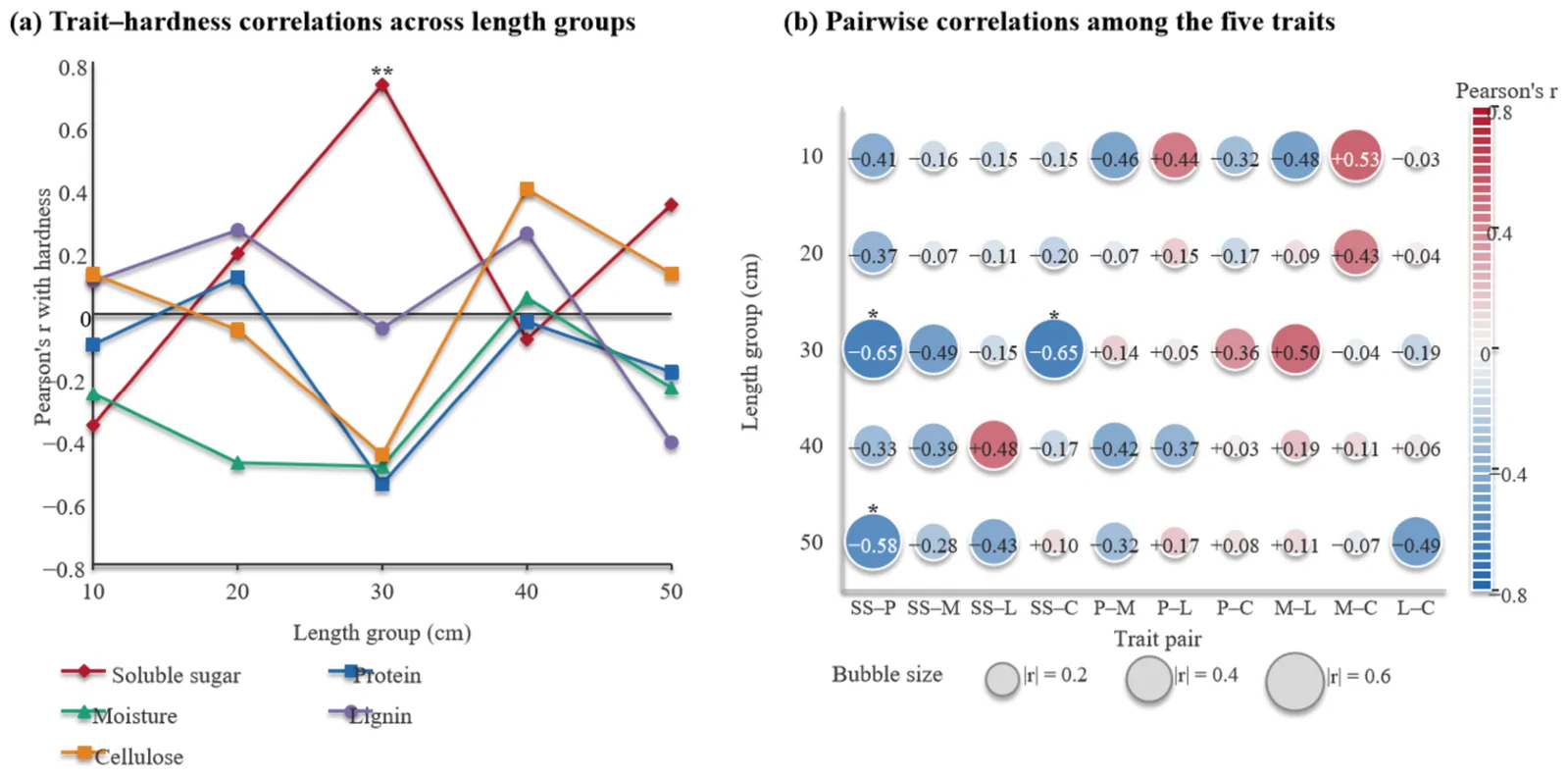
Length-dependent trait–hardness correlations and pairwise intertrait correlations in bamboo shoots. (**a**) Pearson correlation coefficients (r) between each of the five nutritional or structural traits and hardness within the 10-, 20-, 30-, 40-, and 50 cm length classes. (**b**) Pairwise Pearson correlations among soluble sugar, protein, moisture, lignin, and cellulose within each class; bubble color gives the direction of the correlation (red, positive; blue, negative), bubble size is proportional to |r|, and the value inside each bubble is Pearson’s r. *p*-values were adjusted for multiple testing by the Benjamini–Hochberg (BH) procedure, and asterisks mark correlations surviving correction: adjusted *p* < 0.05 (*) and adjusted *p* < 0.01 (**). Sample sizes were *n* = 16, 20, 19, 19, and 22 for the 10-, 20-, 30-, 40-, and 50 cm classes, respectively (total *n* = 96). SS, soluble sugar; P, protein; M, moisture; L, lignin; C, cellulose; BH, Benjamini–Hochberg.

The direction and strength of trait–hardness correlations shifted with length class (Figure 8a). Soluble sugar changed most: r was −0.358 and 0.191 in the 10- and 20 cm classes, rose to 0.728 in the 30 cm class, where it survived BH correction (adjusted *p* = 0.005), and was −0.082 and 0.347 in the 40- and 50 cm classes. The soluble sugar–hardness association in the 30 cm class was thus the strongest of any class.

Other trait–hardness relationships also varied between classes. Moisture was negatively correlated with hardness in the 10-, 20-, 30-, and 50 cm classes (r = −0.256, −0.477, −0.489, and −0.237), most strongly in the 20- and 30 cm classes, and was essentially unrelated in the 40 cm class (r = 0.051). Protein was clearly negatively correlated with hardness in the 30 cm class (r = −0.545) and only weakly elsewhere. Cellulose–hardness correlations were 0.125, −0.052, −0.452, 0.395, and 0.127 in the 10-, 20-, 30-, 40-, and 50 cm classes, respectively, changing sign with class. Lignin was positively correlated with hardness in the 10-, 20-, and 40 cm classes (r = 0.104, 0.266, and 0.255), essentially flat in the 30 cm class (r = −0.047), and negatively correlated in the 50 cm class (r = −0.412).

Overall, trait–hardness structure depended on length class. In the 30 cm class, soluble sugar was strongly positively correlated with hardness, while protein, moisture, and cellulose were moderately negatively correlated. Apart from soluble sugar in that class, no trait–hardness association survived BH correction, so the remainder are trends rather than established relationships.

The five traits yield 10 pairwise combinations, and Figure 8b shows all 50 trait–trait correlations across the five length classes, with bubble color giving the direction of the correlation and bubble size |r|.

Correlation structure differed markedly between classes. In the 10 cm class, the stronger associations centered on moisture: moisture–cellulose was positive (r = 0.527), moisture–lignin and protein–moisture were negative (r = −0.478 and −0.461), and protein–lignin was moderately positive (r = 0.44). Associations were weaker in the 20 cm class, where only moisture–cellulose (r = 0.428) and a negative soluble sugar–protein association (r = −0.373) stood out.

The 30 cm class showed a soluble-sugar-centered pattern. Soluble sugar was strongly negatively correlated with protein and cellulose (r = −0.650 and −0.647), and both survived BH correction; it was also moderately negatively correlated with moisture (r = −0.489), while moisture and lignin were positively correlated (r = 0.50). In the 40 cm class, soluble sugar and lignin were positively correlated (r = 0.481), and protein and moisture were negatively correlated (r = −0.420), with the remaining combinations more scattered.

In the 50 cm class, soluble sugar remained strongly negatively correlated with protein (r = −0.584), again surviving BH correction, and lignin–cellulose (r = −0.491) and soluble sugar–lignin (r = −0.432) showed clear negative trends. Trait covariation therefore reorganized along the length gradient: moisture-centered in the 10- and 20 cm classes, soluble-sugar-centered in the 30 cm class, and more diffuse in the 40- and 50 cm classes. Apart from the associations that survived BH correction, these patterns should be read as trends.

## 4. Discussion

This study compared PLSR, Ridge, and PCA–Ridge for estimating soluble sugar, protein, moisture, lignin, and cellulose in bamboo shoots from 400 to 1000 nm VIS–NIR spectra, tested whether the outer surface and the inner tissue surface differ as spectral sampling regions, and examined how these traits covary with hardness and with one another across shoot length classes. Three findings emerged: prediction accuracy is trait specific, the informative tissue region depends on the trait, and correlation structure reorganizes along the length gradient. We consider each in turn, then set out the limits of what the present data can support.

### 4.1. Model Performance, Spectral Interpretation, and Reliability of Evaluation

In the whole-sample comparison, no single model dominated all traits: PCA–Ridge performed best for soluble sugar and lignin, PLSR for protein, and Ridge for moisture; cellulose was poorly estimated by all models. L2 shrinkage of coefficients for highly correlated bands limits the parameter instability and variance inflation that multicollinearity produces [14], which suits spectra in which adjacent wavelengths are strongly correlated. PCA–Ridge performed best for soluble sugar and lignin in the whole-sample comparison and for soluble sugar from the inner tissue surface in the region-specific analysis. Because PCA reduces dimensionality without reference to the response, its benefit depends on whether trait-related variation lies along the dominant spectral directions; the directions of greatest spectral variance need not coincide with those most relevant to a target trait, so informative but low-variance signal can be attenuated during projection [15]. Comparable hyperspectral studies on agricultural products report that the ranking of modeling methods shifts with sample size, trait distribution, spectral range, preprocessing, and validation design [22,23,24,25]. With modest sample sizes and high collinearity, regularized linear models tend to be the stable choice, whereas nonlinear models generally need more data and more variability before their flexibility pays off [26]. The performance of Ridge for moisture is therefore best read as a good match between regularization and the structure of this trait’s data, rather than as evidence that Ridge is intrinsically superior across traits.

For some traits, test-set R^2^ exceeded training-set R^2^. With a limited sample size, random training and test splits can differ in trait distribution and length-class composition, and screening model and preprocessing combinations against the test set can inflate the apparent result. Nested cross-validation or an independent external test set, keeping model selection, hyperparameter optimization, and final evaluation at separate data levels, would give a more defensible estimate [27,28,29]. Where the data are hierarchical—clumps, individual shoots, and sampling batches—grouping the split by independent biological unit would further limit the effect of related samples appearing on both sides of the split [30]. For this reason, R^2^ and RMSE are reported here as means ± SD across 100 repeated runs, which describes stability more completely than a single value [31].

Physically, the 400–1000 nm window covers the visible (400–700 nm) and short-wave near-infrared (700–1000 nm) regions. Within this range, the dominant molecular absorption is the relatively weak second O–H stretching overtone of water near 970 nm, together with visible pigment absorption and weak third-overtone C–H absorption near 900 nm. The stronger O–H bands at 1200, 1450, and 1940 nm, the C–H second- and first-overtone and combination bands diagnostic of lignin and cellulose at 1150–1250, 1700–1800, and 2100–2350 nm, and the N–H first overtone near 1500 nm all lie outside the measured range. The R^2^ ≈ 0.51 obtained for lignin is therefore more plausibly carried by scattering and tissue-structure covariance with progressive lignification than by direct lignin absorption; the near-zero R^2^ for cellulose is consistent with this physical limit, since cellulose has no strong diagnostic band in the 400–1000 nm window. This window also captures pigment, tissue-structure, and scattering information together, and the O–H absorption of water near 970 nm may contribute to moisture estimation, although no wavelength-importance or loading analysis was performed to confirm which bands drove each model. Near-infrared absorption by organic compounds arises largely from overtones and combination bands of C–H, O–H, and N–H bonds, which are broad and heavily overlapping [7,32], and reflectance from fruit and vegetable tissue is further shaped by scattering from cell arrangement, tissue density, intercellular spaces, and surface condition [33]. Accurate estimation of soluble sugar and moisture therefore probably draws both on signal from the target components and on their covariation with water status, carbohydrate composition, and tissue structure; the same is likely true of protein, lignin, and cellulose. Multi-trait estimation in this window thus rests on an integrated response across many wavelengths and on tissue optical properties, not on single diagnostic absorption features. Preprocessing such as SNV and detrending can strip further nonchemical variation arising from optical path length, scattering, and baseline drift [34]. For a trait as poorly estimated as cellulose, band-selection methods such as CARS and SPA are worth testing to suppress redundant and noisy bands [35,36,37], as is extending acquisition into the 1000–2500 nm region, which carries complementary information on protein and cell-wall components [32,38].

### 4.2. Differences Between the Outer Surface and Inner Tissue Surface and Implications for Region-Specific Detection

Region-specific modeling made the trait dependence explicit. The inner tissue surface provided stronger predictive information for protein and moisture, while the outer surface showed a slight advantage for soluble sugar; for lignin, the regional difference was small, and cellulose showed no consistent advantage from either region. The protein result is the most striking: the best inner-tissue-surface model exceeded the best outer-surface model by about 0.11 in R^2^ (0.62 versus 0.51), which suggests that the tissue ROI samples can substantially influence estimation accuracy for a given trait. For multi-trait detection, then, no single fixed ROI should be assumed to serve every trait; the sampling region should be chosen per trait, or information from several regions combined.

Two mechanisms plausibly underlie these regional differences. Composition differs: water status, cell-wall development, and the spatial distribution of chemical components are not the same at the outer surface as in the inner tissue. Optics differ too: color, roughness, surface structure, and optical path length at the outer surface alter the reflected signal. Because hyperspectral reflectance carries both absorption and scattering information, microstructure and surface condition substantially shape VIS–NIR responses in fruit and vegetable tissue [33,39], and bamboo shoot tissues and developmental stages are known to differ in anatomy, quality traits, and cell-wall development [40,41,42]. Two regions with similar concentrations of a target component can therefore yield different model accuracy. Higher accuracy in one region means its spectrum carries more predictable information about the trait; it does not mean the component is more abundant there.

Practically, this gives a basis for choosing ROIs by target trait: outer-surface information for soluble sugar, inner-tissue-surface information for protein and moisture. Because the inner surface can be imaged only after the shoot is cut, inner-surface models are suited to laboratory screening; for nondestructive use, the regional comparison mainly indicates which traits outer-surface spectra can support and where additional information would be needed. Modeling both regions of the same shoot would then test whether fusing the two improves accuracy and robustness beyond either alone.

### 4.3. Length-Class Dependence of Trait–Hardness Relationships

Trait–hardness relationships were not stable across length classes. In the 30 cm class, soluble sugar was positively correlated with hardness and survived BH correction (r = 0.728, adjusted *p* = 0.005), indicating strong covariation at that stage. Protein, moisture, and cellulose were negatively correlated with hardness in the same class (r = −0.545, −0.489, and −0.452) but none survived correction, and the positive cellulose–hardness association in the 40 cm class and the negative lignin–hardness association in the 50 cm class are best treated as trends. Texture in fruit and vegetables is governed jointly by cell turgor, intercellular adhesion, and cell-wall composition [43,44], with cell-wall growth and structural change further modulating mechanical properties [45]. Bamboo shoot hardness is therefore a composite phenotype set by water status, cell-wall deposition, tissue anatomy, and the pace of growth.

Postharvest studies of bamboo shoots generally report hardness, lignin, and cellulose rising together as storage and lignification proceed [46,47,48], and near-infrared spectroscopy has been used to predict shoot hardness nondestructively [9]. The present study, however, sampled shoots at different lengths during active growth rather than during postharvest senescence. That the 30 cm class showed a positive soluble sugar–hardness correlation and a negative cellulose–hardness trend suggests that texture during rapid elongation is not formed the same way as during postharvest lignification. During elongation, puncture hardness is likely dominated by tissue water status, cell expansion, and developmental stage [40,43], whereas cell-wall remodeling and lignin deposition become more important after harvest. Soluble sugars, meanwhile, serve osmotic regulation and respiration and supply carbon for structural synthesis, so sugar content and hardness may both track developmental progression rather than one driving the other. Correlation directions observed along a growth gradient should not, therefore, be transferred to postharvest storage. Measuring tissue turgor, cell-wall thickness, lignification staining, and the relevant enzyme activities on the same shoots would separate the contributions of water status, structural deposition, and carbon metabolism. Because the present design is cross-sectional—different shoots sampled once across length classes rather than the same shoots tracked over time—the length-class patterns should be read as developmental-stage associations, not as a longitudinal time series.

### 4.4. Length-Class-Specific Trait Correlation Structures and Their Biological Implications

Correlation structure also reorganized along the length gradient. Associations in the 10- and 20 cm classes centered on moisture, consistent with early tissue expansion and water status providing the dominant background for trait covariation. In the 30 cm class, soluble-sugar-related combinations came to the fore: soluble sugar was negatively correlated with protein and cellulose (r = −0.650 and −0.647) and positively correlated with hardness, all surviving BH correction. In the 50 cm class, the negative soluble sugar–protein correlation persisted (r = −0.584). This is consistent with what is known of shoot development: sugars and other components change with growth stage and tissue region [40], accompanied by progressive cell-wall formation and lignification [41,49], and carbohydrate and structural-polysaccharide metabolism continue to shift after harvest as lignification advances [46,50]. Soluble sugars act both as carbon and energy substrates and as signals regulating growth, development, and gene expression [51,52]; shoots are rich in protein, carbohydrates, minerals, and dietary fiber [53]; and rapid bamboo growth demands coordinated water and nutrient uptake, assimilate transport, and biomass allocation [54,55,56]. Chromosome-scale bamboo genome assemblies have further linked cell-wall biosynthesis gene families to shoot development, providing molecular context for structural-material formation during rapid growth [57]. The sugar–protein and sugar–cellulose associations reported here are nonetheless statistical covariation: they cannot establish the direction of carbon flow or show that one trait promotes or suppresses another. They are better read as candidate leads for coordinated adjustment between carbon metabolism and structural-material formation across shoot length classes.

### 4.5. Study Limitations and Future Perspectives

Three constraints bound what these results can support: sample composition, the spatial correspondence between reference measurement and ROI, and the validation design. On composition, the study centered on *D. brandisii* grown under protection in Fuyang, Zhejiang, but also included shoots of other bamboo species from a field nursery in Anji as reference material. Because these reference shoots entered model construction, the models describe spectral–trait relationships in a mixed data set rather than within *D. brandisii* alone. Fitting a within-species model and a joint model on additional independent *D. brandisii* shoots would separate within-species accuracy from cross-material generalization. As a sensitivity check, we refit the region-specific models after excluding the six reference-variety shoots (IDs c1–c6). Test-set R^2^ and RMSE values were essentially unchanged (differences <0.01 in R^2^ and <0.05 in RMSE), indicating that the inclusion of a small number of reference shoots had a negligible effect on model performance.

Spatial correspondence between the reference measurement and the ROI governs reproducibility. The definitions of outer surface and inner tissue surface, and the position, area, and pixel count of each ROI, should follow explicit criteria, and the spectral sampling region should match the tissue actually used for the corresponding wet-chemistry measurement as closely as possible. Across batches and sites, reflectance calibration should be standardized and the time elapsed since excision, surface condition, and instrument status controlled, so that spectra remain comparable between acquisitions.

Validation design determines how much the reported accuracies mean. The present evaluation rests on repeated internal random splits; independent validation or stricter resampling would assess generalization more objectively [29,31]. Given the hierarchical structure of the data—clumps, individual shoots, sampling batches—splitting by independent biological unit would reduce leakage from related samples falling on both sides of the split [24]. Model selection and hyperparameter optimization belong in an inner cross-validation loop, with outer folds reserved for evaluation and for generating out-of-fold (OOF) predictions [27,28]; where predictions feed downstream correlation analysis, independent test-set or OOF predictions should be preferred over predictions from training fits, which are optimistically biased. Within these limits, the results support VIS–NIR hyperspectral imaging as a feasible route to multi-trait estimation and tissue-region optimization in bamboo shoots. Validating on independent *D. brandisii* shoots across sites, batches, and instruments is the necessary next step toward standardized rapid detection, and calibration transfer or domain adaptation [58,59], multi-region or multisource fusion [60], and portable spectrometers [61] are the obvious technical routes. Expanding the number of independent biological replicates and clarifying sample-level hierarchy would strengthen inference, while pairing spectra with histology, cell-wall structure, and metabolic phenotypes would connect spectral differences to cell-wall development, carbon metabolism, and texture [62]. The added value of longer-wavelength NIR information and of multi-region spectral fusion remains to be quantified.

## 5. Conclusions

VIS–NIR hyperspectral imaging over 400–1000 nm, combined with regularized regression, showed potential for estimating several nutritional and structural traits of bamboo shoots from a single acquisition. Among PLSR, Ridge, and PCA-Ridge, no single model was uniformly best across traits; PCA-Ridge performed best for soluble sugar and lignin, PLSR for protein, and Ridge for moisture. Cellulose remained difficult to predict reliably.

The tissue region sampled matters, and it matters differently for each trait. The inner tissue surface provided stronger predictive information for protein and moisture, while the outer surface showed a slight advantage for soluble sugar; for lignin, the difference between regions was small. Choosing the ROI by target trait therefore may improve the specificity of multi-trait detection, and combining regions is a natural next step.

Across length classes, relationships among nutritional traits, structural traits, and hardness were not stable. The 30 cm class stood out, with soluble sugar positively correlated with hardness and negatively correlated with protein and cellulose, pointing to concentrated covariation among nutritional, structural, and textural traits at that stage and offering candidate leads for future work on trait coordination during shoot growth.

These conclusions rest on repeated internal data splits and on models that included reference shoots of other bamboo varieties. Independent *D. brandisii* samples, and validation across sites, batches, and instruments, are needed before the models can be considered stable or transferable.

## Data Availability

The hyperspectral images and reference trait measurements that support the findings of this study are available from the corresponding author upon reasonable request.
